# Limited intimal tear of the ascending aorta in a young patient: a case report

**DOI:** 10.1186/s13019-026-04140-7

**Published:** 2026-04-24

**Authors:** Ryota Nomura, Takahiro Suzuki, Muneaki Yamada, Masanao Nakai

**Affiliations:** Department of Cardiovascular Surgery, Shizuoka City Shizuoka Hospital, Shizuoka, 10-93 Otemachi, Aoi-ku, Shizuoka, 420-8630 Japan

**Keywords:** Limited intimal tear, Acute aortic syndrome

## Abstract

A 34-year-old man developed sudden retrosternal pain during exercise. Initial CT suggested type A dissection without a visible flap, showing mild pericardial effusion, ascending aortic dilatation, and a subtle bulge. Follow-up CT on day 3 revealed progression, prompting urgent valve-sparing aortic root replacement. Intraoperative findings confirmed a linear intimal fissure. Histology showed cystic medial necrosis. Diagnosis of limited intimal tear (LIT) is challenging due to subtle radiologic signs. Clinicians should suspect this lesion in patients with compatible symptoms despite inconclusive imaging, as prompt surgery is essential to prevent rupture.

## Background

Acute aortic syndrome is a potentially fatal disease, and it is crucial to determine whether to perform emergency surgery or to pursue conservative management. When deciding on a treatment policy, the results of contrast-enhanced computed tomography (CT) are considered important in daily clinical practice. In majority of cases, extensive intramedial dissection resulted in the double-barrel lumen of classic dissection; in other cases, however, there was no significant dissection into the media, which is referred to as incomplete tear, or limited, subtle or discrete dissection.

Herein, we describe an unusual clinical presentation of aortic dissection in a 34-year-old male patient who presented with atypical aortic contour bulging and a linear intimal fissure in the ascending aorta and subsequently underwent urgent surgery, consisting of valve sparing aortic root replacement.

## Case presentation

**Fig. 1:**
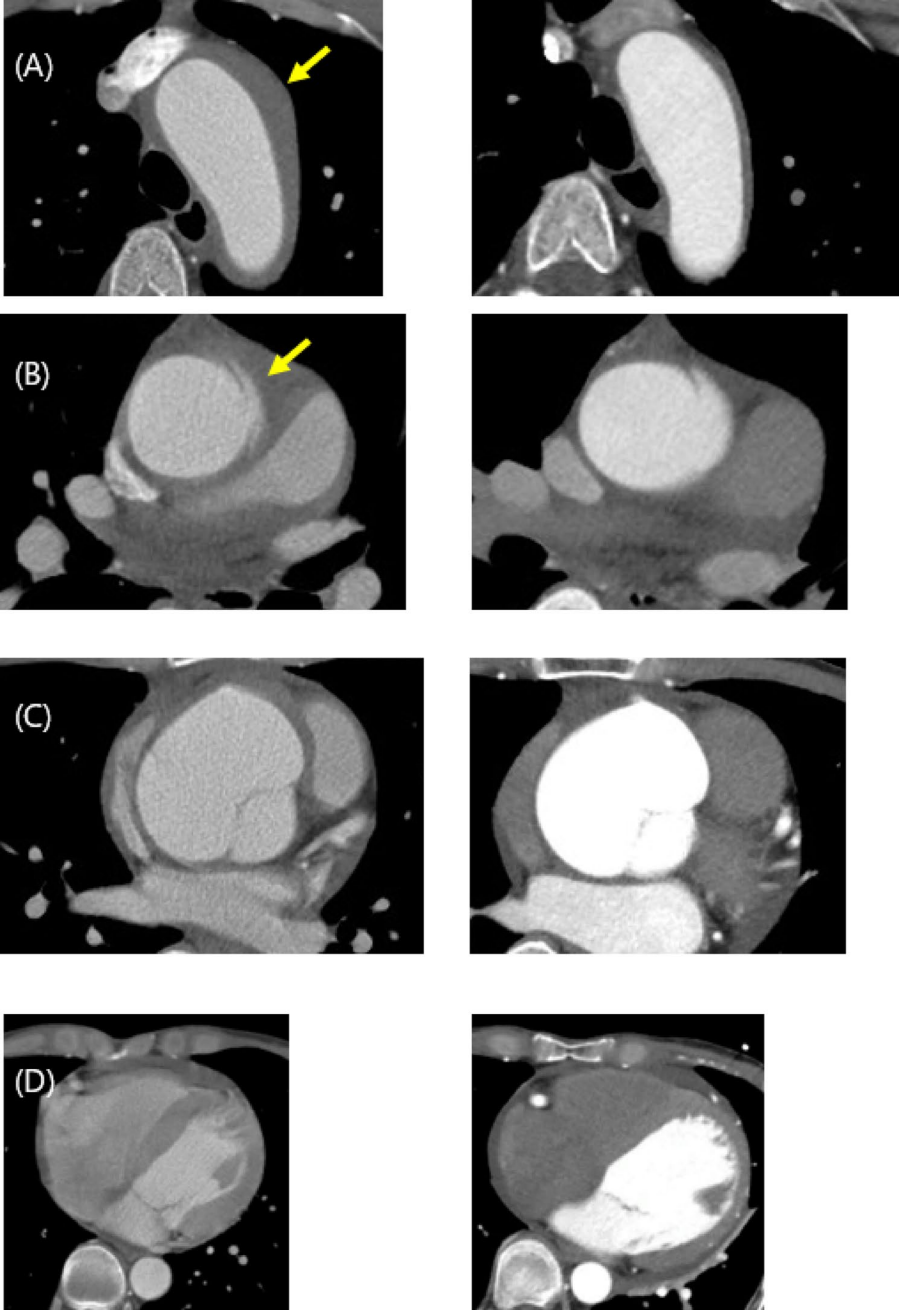
Comparison between initial and follow-up contrast-enhanced CT images. The left column shows the initial CT at presentation, and the right column shows the follow-up CT obtained on day 3 at corresponding anatomical levels. (**A**) Blood tracking along the aortic arch on the initial CT, (arrow), which resolved on follow-up imaging., (**B**) Serial axial images demonstrating progression of the aortic contour abnormality in the proximal ascending aorta; a subtle contour bulge on the initial CT, (arrow) becomes more conspicuous on follow-up imaging., (**C**) Dilatation of the aortic root measuring 60 mm, without significant interval change., (**D**) Mild pericardial effusion, which remained stable on follow-up imaging

A 34-year-old male with no remarkable medical history was brought to the emergency department of a peripheral hospital because of moderate chest pain during vigorous exercise, described as a sudden, stabbing retrosternal pain. As part of the diagnostic workup for acute thoracic pain, computed tomography (CT) was performed, which suggested type A aortic dissection without a clearly identifiable intimal tear. The patient was transferred to our department and arrived hemodynamically stable, with resolution of symptoms. A 12-lead electrocardiogram showed no ST-T changes. Echocardiography revealed mild pericardial effusion and moderate aortic regurgitation with a central jet in the setting of preserved left ventricular size and systolic function (left ventricular end-diastolic/systolic diameters: 53/33 mm; ejection fraction: 68%). All laboratory results were within normal limits, including white blood cell count, liver enzymes, renal function, and D-dimer levels. Emergent contrast-enhanced CT showed mild anterior localized pericardial effusion, dilatation of the aortic root measuring 60 mm and the ascending aorta measuring 40 mm, and blood tracking along the aortic arch, without a clearly identifiable intimal flap. There was also a subtle contour bulge along the medial aspect of the proximal ascending aorta (Fig. 1). Given these findings, the primary differential diagnoses included intramural hematoma and thrombosed type A aortic dissection. Other potential causes of acute chest pain, such as acute coronary syndrome and pulmonary embolism, were considered unlikely based on electrocardiographic findings, laboratory data, and imaging studies. The patient was hospitalized and placed under observation.

**Fig. 2:**
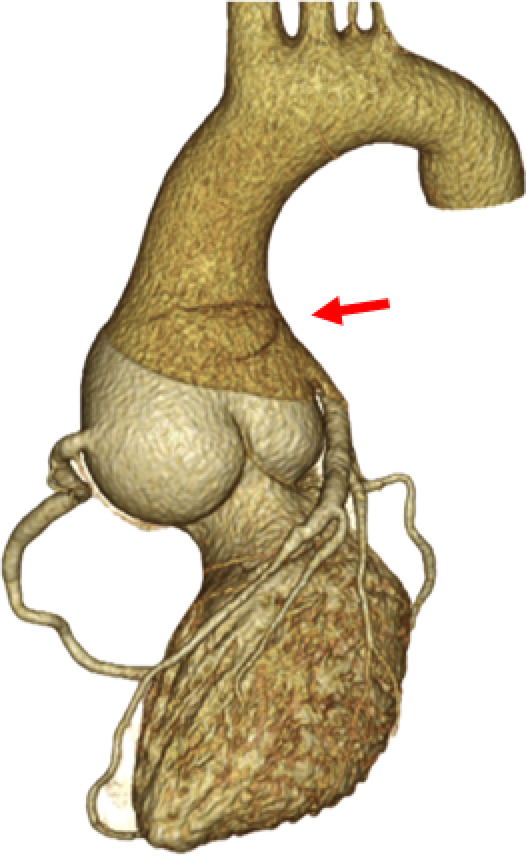
Three-dimensional reconstruction from follow-up CT. The intimal tear in the proximal ascending aorta is clearly demonstrated (arrow), corresponding to the lesion identified on axial CT images

Following admission, the patient was managed in the intensive care unit under strict blood pressure control with multiple antihypertensive agents and close hemodynamic monitoring. The patient remained asymptomatic throughout hospitalization. Serial CT imaging was performed. As the initial findings were not definitive for classic type A dissection and the patient was hemodynamically stable without progression of pericardial effusion, a strategy of close observation with repeat imaging was adopted. Follow-up CT performed on the third day of hospitalization more clearly demonstrated the intimal tear in the proximal ascending aorta. Three-dimensional reconstruction further clarified the lesion. Blood tracking along the aortic arch had resolved, and there was no increase in pericardial effusion. The lesion became more conspicuous on follow-up imaging (Figs. 1 and 2). Given these findings, we decided to proceed with urgent surgical intervention. Because of significant aortic root dilatation, aortic root replacement was indicated.

**Fig. 3:**
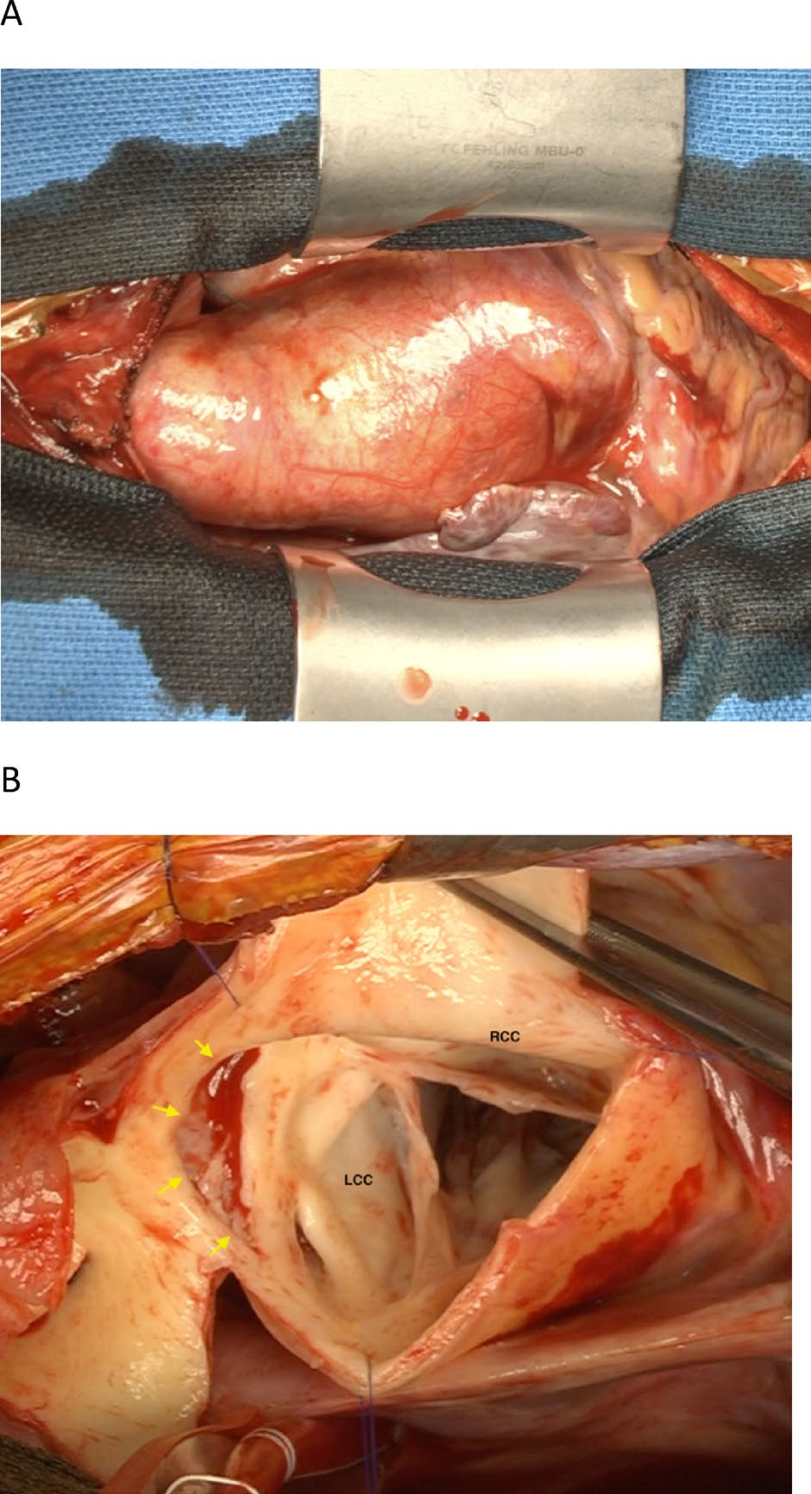
Intraoperative views showing aortic root dilatation and a linear intimal tear. Intraoperative view shows aortic root dilatation (**A**) and an extensive linear intimal tear seen from the luminal side of the aorta (arrows) (**B**)

Surgery was performed through a midline sternotomy. Cardiopulmonary bypass was established with single arterial cannulation via the right femoral artery and venous drainage through the right atrium. Dense adhesions between the ascending aorta and the pulmonary artery precluded safe dissection. After confirming the absence of dissection in the distal ascending aorta by epiaortic echocardiography, the ascending aorta was cross-clamped, and cardiac arrest was achieved with retrograde cardioplegia, followed by intermittent selective antegrade and retrograde cold blood cardioplegia after aortotomy. The aorta was then opened to allow direct inspection of the aortic root. Linear intimal fissure and aortic wall thickening in the proximal ascending aorta were confirmed (Fig. 3). Cusp inspection revealed normal area and height of the cusps. Given the patient’s young age and favorable cusp morphology, valve-sparing aortic root replacement using the reimplantation technique was performed. Histologic tests revealed cystic medial necrosis of the aortic wall. Postoperative echocardiography showed trivial residual aortic regurgitation. The patient was discharged on postoperative day 15 without any complications.

## Discussion

In recent years, imaging technology has advanced remarkably, and in the diagnosis of aortic dissection, sensitivities and specificities as high as 98% have been reported [1]. Despite this data, a recent study of patients with Medicare claims determined that 4.5% of aortic dissections were missed in the emergency department [2, 3].

This diagnostic challenge was already recognized in 1999, when Svensson et al. [4] described nine cases of aortic dissection that went undetected on various imaging modalities. To address this issue, they proposed a new classification system for aortic dissection variants, differentiating an eccentric ascending aortic bulge—designated as “class 3” dissection—from the classic intimal flap and intramural hematoma types; class 1, classic dissection is a well-recognized form of aortic dissection and is characterized by a flap between true and false aneurysm and clot in false lumen; class 2, intramural hematoma is less common, and the dissection is usually filled with blood clot without a detectable intima tear; class 3 dissection is characterized by limited stellate or linear intimal tear associated with exposure underlying aortic media or adventitial layers with eccentric bulge at tear site, but without the progression and separation of the medial layers; class 4 dissection is a penetrating atherosclerotic ulcer with surrounding hematoma, usually subadventitial; class 5 dissection is iatrogenic or traumatic dissection illustrated by coronary catheter causing dissection. Even though class 3 dissection variant has since been widely adopted, most physicians remain unfamiliar with this lesion [5].

Herein, we describe a challenging case of class 3 dissection that was not definitively diagnosed at initial presentation. Although the patient presented with acute chest pain, a mildly aneurysmal ascending aorta, and a small pericardial effusion, the limited intimal tear (LIT) was not identified at the time of initial evaluation. A follow-up CT performed three days later more precisely demonstrated the extent and location of the intimal tear in the proximal ascending aorta, leading to the decision to proceed with urgent surgery. The lesion corresponded approximately to Ishimaru zone 0, although this classification is primarily used in the context of endovascular repair. According to the GERAADA score, the patient was categorized as low surgical risk. Together with the absence of definitive imaging findings at initial presentation, this contributed to the initial decision to pursue close observation with planned early re-evaluation. In retrospect, the subtle contour bulge observed on the initial CT (Fig. 1B) may represent a localized dissection or contrast extravasation rather than simple aortic wall bulging. Although these findings, together with the presence of pericardial effusion, could have warranted immediate surgical intervention, the diagnosis was not definitive at the time of initial evaluation. The patient was therefore managed with close observation and serial imaging.

This case highlights the inherent diagnostic difficulty of limited intimal tear, even among experienced clinicians, particularly when imaging findings are subtle and not accompanied by a clearly identifiable intimal flap. In class 3 dissection, the only layer covering the aortic lumen is extremely thin and therefore prone to rupture. Consequently, this type of dissection should be managed in the same manner as classical aortic dissection. Clinicians involved in the diagnosis and management of acute aortic syndrome must remain vigilant for this variant, particularly when clinical suspicion is high but no obvious aortic lesion is detected on imaging, as delayed recognition may lead to catastrophic outcomes, including fatal rupture.

## Data Availability

No datasets were generated or analysed during the current study.
